# Gefitinib is more effective in never-smokers with non-small-cell lung cancer: experience among Asian patients

**DOI:** 10.1038/sj.bjc.6602652

**Published:** 2005-06-07

**Authors:** S-T Lim, E-H Wong, K-L Chuah, S-S Leong, W-T Lim, M-H Tay, C-K Toh, E-H Tan

**Affiliations:** 1 Department of Medical Oncology, National Cancer Centre, 11 Hospital Drive, Singapore 169610, Singapore; 2 Division of Clinical Trials and Epidemiological Sciences, National Cancer Centre, Singapore, Singapore; 3 Department of Pathology, Singapore General Hospital, Singapore, Singapore

**Keywords:** gefitinib, non-small-cell lung cancer, never-smoker

## Abstract

We retrospectively analysed the results of patients with advanced non-small-cell lung cancer treated with gefitinib to derive clinical factors predictive of response and a favourable survival outcome. Patients were treated with gefitinib 250 mg per day and re-evaluated 4–8 weeks later with repeat CT scan and every 8 weeks thereafter to assess response and the duration of response. Pathology review by a histopathologist was conducted, in particular to confirm a recently published result of bronchioloalveolar carcinoma histology or its components as predictive of response to gefitinib. Logistic regression and Cox regression analytical methods were applied to determine factors that could predict for response and improved overall survival. A total of 110 patients were treated. The overall response rate was 32% partial responses (PRs). Only never-smoking status was predictive of response in the logistic regression analysis, adjusted OR=6.1, 95% CI=1.7, 21.5. The presence of a PR and good performance status were predictive of a favourable survival outcome from the Cox regression modelling. Responders had an adjusted HR of 3.0, 95% CI=1.5–5.8 compared to nonresponders, while patients with ECOG status 0–1 had an adjusted HR of 0.42, 95% CI=0.25–0.72, compared with patients with ECOG status 2–4. Bronchioloalveolar carcinoma or its components were distinctly absent on pathology review. In conclusions, Never-smoking status is an important clinical predictor of a favourable response to gefitinib.

The treatment of advanced non-small-cell lung cancer (NSCLC) has seen significant advances over the past two decades. Platinum-based regimens became established treatment due to the survival advantage shown despite the modest response rates and short duration of activity (Non-small Cell Lung Cancer Collaborative Group, 1995). The second-generation agents such as vinorelbine, gemcitabine, and taxanes have made further strides in this regard (Bunn, 2002). However, further development of cytotoxics seems to have hit a snag and it is the general feeling that this has peaked and hence unlikely to cross the next hurdle.

Targeted therapy has now taken centre stage in cancer therapeutics development. The success of trastuzumab (Herceptin) and ST1571 (Gleevec) in the treatment of breast cancer and gastrointestinal stromal tumours respectively heralded a new era in solid tumour therapy (Slamon *et al*, 2001; Demetri *et al*, 2002). Gefitinib (Iressa) became the first targeted form of treatment available for treatment of NSCLC. The well-reported phase II studies (IDEAL I and 2 trials) have shown encouraging responses (8.8–19% response rates) even in patients who have been heavily pretreated (Fukuoka *et al*, 2003; Kris *et al*, 2003).

Gefitinib first became available in Singapore in December 2000 through Astra-Zeneca's Expanded Access Program and was subsequently approved for use as salvage treatment for patients with advanced NSCLC in the country in May 2003. We first reported the clear superior activity of gefitinib among patients who were never-smokers at the 39th American Society of Clinical Oncology annual meeting. This report is a follow-up on an expanded group of patients who were treated with gefitinib. The purpose of this retrospective analysis was to assess the response to gefitinib and to identify clinical parameters that may predict for response to gefitinib. Being better able to select appropriate patients for therapy would translate to better resource utilisation especially since treatment using these novel agents is extremely costly. In addition, this might form the basis for the identification of a set of genetic parameters that can further predict gefitinib sensitivity.

## PATIENTS AND METHODS

Between January 2001 to October 2003, 110 patients with metastatic NSCLC treated with gefitinib as monotherapy were retrospectively reviewed. Among them, 33 patients were treated on the Expanded Access Clinical Program. This cohort of 110 patients came from a series of approximately 600 patients with NSCLC seen in our department over the same time period. Most of patients (92%) have failed at least one line of chemotherapy. Follow-up and mortality information were available for all patients.

All patients had baseline CT scans, full blood count, liver and renal biochemistries prior to treatment with gefitinib. CT scans were performed 4–8 weeks after treatment to assess for response according to World Health Organisation (WHO) standard criteria. Stable disease (SD) and partial response (PR) were confirmed on repeat CT scans not earlier than 4 weeks after the first evaluation CT scan. Complete blood counts, renal biochemistries, and liver biochemistries were repeated at 4–8 weeks intervals. Our centre's radiologists reviewed all CT evaluations. Other clinical information such as age, sex, smoking history, histology, ECOG performance status, and number of previous lines of chemotherapy prior to gefitinib, were retrospectively reviewed and analysed for possible association with response. Never-smoker was defined as one who had never smoked at all prior to diagnosis of NSCLC. A smoker was defined as one who was a current or ex-smoker.

We performed pathology review of the patients diagnosed in our institutions. The thoracic histopathologist (KLC) was blinded to the response outcome of the patients and was required to re-classify the histology of the slides of the patients. In particular, he was required to look specifically for bronchioloalveolar components during the review, using the criteria described by Ebright *et al* (2002) in their study.

### Statistical methods

The following variables were included in a logistic regression model to investigate their association with response – gender, smoking history, ECOG performance status, age, number of lines of prior chemotherapy, and histology. These factors, in addition to response were investigated for their influence on survival using Cox regression. All variables were entered simultaneously in both models. Variables with *P*-value of less than 0.05 were considered statistically significant. All analyses were performed using SPSS version 10.0 (SPSS Inc., Chicago, IL, USA).

## RESULTS

The patient demographics are listed in Table 1. The median age of patients when gefitinib was started was 55.8 years. In total, 53 and 60% of the patients were females and lifetime nonsmokers, respectively. Majority of the patients (93%) were of Chinese origin. Most of our patients had received at least two lines of chemotherapy (71%) prior to starting gefitinib. The majority of the patients were diagnosed with adenocarcinoma (71%). Only one patient had bronchioloalveolar carcinoma. Pathology review did not reveal any bronchioloalveolar carcinoma or such components in those patients diagnosed in our institutions (Table 2). The slides of 25 patients whose diagnoses were made elsewhere were not available for review. The sole patient originally diagnosed to have bronchioloalveolar carcinoma was reclassified as adenocarcinoma.

The overall observed PR rate was 32% (Table 3). There were no complete responders (CR). Partial responses were, however, experienced more commonly among patients who were of the female gender (43 *vs* 19%), and lifetime never-smokers (47 *vs* 9%). More partial responders were seen among female never-smokers than their male counterpart (52 *vs* 33%), but this difference was not statistically significant.

Using logistic regression, the following potential prognostic factors were investigated for possible association with the response outcome: gender, smoking history, age at diagnosis, number of lines of prior chemotherapy, performance status using the ECOG (Eastern Cooperative Oncology Group) scale, and histology. Only smoking history turned out to be independently predictive of response to gefitinib after adjusting for the other variables (Table 4). Never-smokers had a 6.1 (95% CI, 1.7–21.5) higher odds of response compared to smokers.

**Figure 1:**
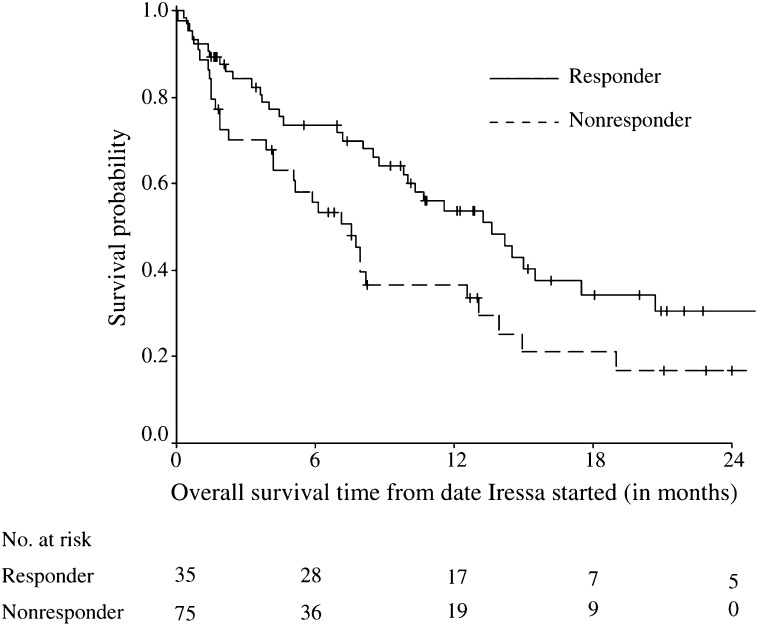
Kaplan–Meier estimates of overall survival time comparing responders (CR/PR) and nonresponders (SD/PD).

**Figure 2:**
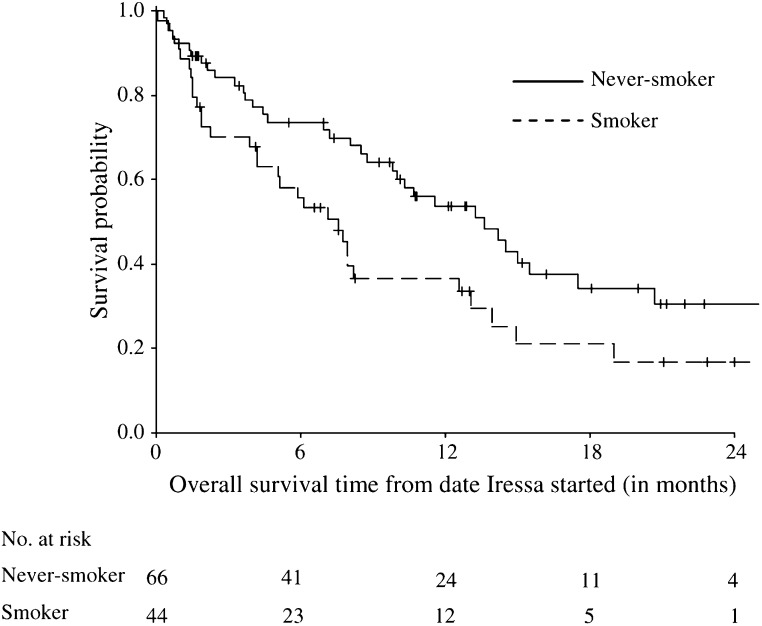
Kaplan–Meier estimates of overall survival time comparing never-smokers and smokers.

Overall survival was defined as the period from the date a patient was started on gefitinib to the last follow-up date (censored) or date of death. Figures 1 and 2 show the Kaplan–Meier estimates of overall survival comparing responders (PR) with nonresponders (SD/PD) and never-smokers with smokers, respectively, not adjusting for any other variables. Responders had a longer median overall survival compared to nonresponders (17.5 *vs* 7 months, HR=3.0, 95% CI=1.65–5.59). Never-smokers were found to have a better overall survival than smokers (median survival 13.6 *vs* 7.6 months, HR=0.61, 9% CI=0.38, 0.99).

The potential prognostic factors used in the logistic regression earlier and the type of responses (PR *vs* SD/PD) were also investigated in a Cox regression model to determine independent predictive factors for improved survival outcome (Table 5). Of the seven variables included, the presence of a PR and good ECOG status were found to be predictive of improved survival. Patients with SD/PD had almost three times (95% CI 1.54–5.77) higher risk of death compared to those with PR while those with good ECOG status had 0.42 times lower risk of death (95% CI 0.25–0.72) compared with the other group, adjusting for the other variables found in the table. Smoking history was not found to be significant in explaining survival, with an adjusted HR=0.88 and 95% CI=0.48–1.63. Similar results were obtained when overall survival was redefined as the period from date of diagnosis till the last follow-up (censored) or date of death (Table 6).

Gefitinib was very well tolerated in our patients (Table 7). The most common toxicity was rash, occurring in 38 (34.5%) patients. However, the majority of patients had grade 1 and 2 toxicity. Other common toxicities include conjunctivitis, diarrhoea, and elevation of the serum transminases.

## DISCUSSION

Gefitinib is the first targeted therapy proven to work and approved for use as salvage treatment in advanced NSCLC. The response rates in the salvage setting as obtained from IDEAL 1 and 2 studies are comparable to that of docetaxel, which is currently the standard of care for second-line use (Shepherd *et al*, 2000; Fukuoka *et al*, 2003; Kris *et al*, 2003). However, its superior toxicity profile and convenient oral dosing form stand out as definite advantages when compared to cytotoxics. Its targeted and selective inhibition of HER1/EGFR tyrosine kinase and hence the downstream pathway(s) driving and maintaining carcinogenesis accounts for its low toxicity and for its non-cross resistance with cytotoxics. Our study and others have shown that previous exposure to chemotherapy did not have an effect on subsequent response to gefitinib (Haringhuizen *et al*, 2004; Miller *et al*, 2004b). Its specificity for a particular target also implies the possibility of using a biomarker to predict for response, similar to HER2 status predicting for response to trastuzumab in breast cancer (Yu and Hung, 2000). Despite the obvious target of gefitinib, expression of EGFR is not predictive for response to gefitinib (Bailey *et al*, 2003). Until recently, no biomarkers or clinical features were found to be predictive for response to gefitinib. For this reason, the INTACT investigators were unselective in the patient populations for their studies and this failure to adequately select for appropriate patients probably led to a negative outcome in contradistinction to the trastuzumab study in metastatic breast cancer (Giaccone *et al*, 2004; Herbst *et al*, 2004).

Our initial experience with gefitinib in patients with advanced NSCLC after failing multiple lines of cytotoxics intrigued us in that we noticed that the responders were mainly those who were never-smokers. It is of interest that others also had similar experiences (Haringhuizen *et al*, 2004; Miller *et al*, 2004b). It is known that there are significant differences in the mutational frequencies and spectra in lung cancers between smokers and nonsmokers. For instance, p53 and K-ras mutations were detected more frequently in smokers with lung cancer (Ahrendt *et al*, 2000; Gealy *et al*, 2001). That would imply that the predominant pathways driving and/or maintaining the carcinogenic process might be different between these two groups of patients. Sanchez-Cespedes *et al* (2001) and Wong *et al* (2002) used microsatellite analysis to characterise and to determine the frequency of loss of heterozygosity/allelic gains in adenocarcinoma of the lung from smokers/ex-smokers and nonsmokers and found distinct differences between the two groups. Although the results of both studies were somewhat discrepant, it is clear that the genetic makeup of adenocarcinoma from smokers and nonsmokers is different. It is conceivable that different genetic pathways are involved in carcinogenesis between these two groups from these studies.

We looked for possible differences in outcome between smokers and nonsmokers in patients with advanced NSCLC treated with platinum-based chemotherapy in our previous study (Toh *et al*, 2004). In that study that included 317 patients (115 patients were nonsmokers), no differences were observed in terms of response to cytotoxics and survival outcome between smokers and nonsmokers despite distinct differences in age at diagnosis, and histology, and gender distribution. Given that cytotoxics kill cancer cells in a nonselective manner in that they do not target specific molecular pathway(s), it can be difficult to appreciate the impact of any mutational differences between these two groups of patients with the use of chemotherapy. Gefitinib, a more targeted form of treatment that inhibit the predominant pathway(s) that initiate and maintain the carcinogenic process may make this biological difference between tobacco-induced and nontobacco induced lung cancer more manifest. This is in line with the observations made in this and other studies as well (Yu and Hung, 2000; Haringhuizen *et al*, 2004).

The recent discovery of somatic activating mutations in the tyrosine kinase domain of the EGFR gene in the tumour brings new hope of improved ability to predict response to gefitinib (Lynch *et al*, 2004; Paez *et al*, 2004). These two studies found that these mutations predicted well for gefitinib response. However, both studies included small numbers of patients treated with gefitinib. In addition, Paez *et al* found that these mutations were present more frequently in adenocarcinomas, female gender, and patients from Japan in a cohort of 119 patients who were not treated with gefitinib. It was not established in this study whether the mutations correlated with nonsmoking status as well although Lynch *et al*'s (2004) study does suggest indirectly that a correlation with nonsmoking status was found. Lynch *et al* also sequenced the entire coding region of the EGFR gene in tumours from 25 patients with NSCLC not treated with gefitinib, including 15 patients with bronchioloalveolar carcinoma, and found these mutations in two patients, both of whom had bronchioloalveolar carcinoma. These findings are certainly intriguing and will need confirmation in a larger study population. Further studies will need to be done to confirm if Asian and Caucasian patients differ significantly in this aspect. If the latter proves to be so, it implies that gefitinib would benefit Asian patients more than Caucasian patients.

Miller *et al* (2004b) reported that the presence of bronchioloalveolar component as an independent predictor of gefitinib responsiveness. However, this finding was not observed in our study. Only nonsmoking status was predictive of response to gefitinib in our cohort. Similarly, in a recent report from Korea involving 90 patients with NSCLC treated with gefitinib, histology was not found to be an independent predictor for response to gefitinib (Han *et al*, 2005). Of importance, the frequency of EGFR mutation in patients with bronchioalveolar carcinoma was similar to that seen in patients with non-BAC adenocarcinomas (Han *et al*, 2005). Thus, it remains to be established if bronchioloalveolar histology is associated with a distinct mutational spectrum that is different from the other histological entities. Moreover, the classification of variants of bronchioloalveolar histology suffers from interobserver differences and reproducibility. This coupled with the rarity of this histological type and its subtypes further reduced its usefulness as a predictive factor for response to gefitinib.

Until a confirmed biomarker for response to gefitinib is routinely and readily available, an economical and simple way to select for appropriate patients for this expensive form of treatment is the smoking status. Based on the relatively higher incidence of never-smokers diagnosed with lung cancer especially among women in Asia, it does suggest that a higher proportion of patients in this part of the world may benefit from treatment with gefitinib (MacLennan *et al*, 1977; Koo *et al*, 1985; Gao *et al*, 1987; Han *et al*, 1990). Our previous study revealed that 36% of patients referred to our department were never-smokers (Toh *et al*, 2004). Despite the negative results from the INTACT studies, it is still worthwhile exploring the use of chemotherapy with gefitinib in never-smoking patients with advanced NSCLC. The TRIBUTE study, which compared erlotinib (Tarceva) with placebo in patients with advanced NSCLC receiving paclitaxel and carboplatin as first-line therapy was reported at the 2004 Annual Meeting of the American Society of Clinical Oncologist (Miller *et al*, 2004a). The design and the final results of this study were similar to the INTACT studies. However, a predetermined analysis of the subset of patients who never smoked showed a significantly improved median survival in those randomised to received erlotinib (23 *vs* 10 months; HR 0.49, CI 0.28–0.85).

It is important that there should be continuing efforts at genetic profiling of lung cancer patients in Asia to allow correlation with treatment response especially to targeted agents. Such a prospective study is ongoing in this centre.

## Figures and Tables

**Table 1:** Patient demographics

**Characteristic**	**Number**	**%**
*Sex*		
Male	52	47
Female	58	53
		
*Race*		
Chinese	102	93
Malay	5	5
Indian	3	3
		
*Age when Iressa started (years)*		
Median (interquartile range)	55.8 (15.1)	
Min, max	36.7, 92.1	
		
*Smoker*		
No	66	60.0
Yes	44	40.0
		
*Histology*		
Adenocarcinoma	78	71
Squamous cell carcinoma	11	10
Large cell/undifferentiated	12	11
Bronchioloalveolar carcinoma	1	1
NSCLC, NOS	8	7
		
*Performance status (ECOG)*		
0–1	79	72
2–4	31	28
		
*No. of previous lines of chemotherapy*		
0	9	8
1	23	21
2 or more	78	71

**Table 2:** Pathology review *vs* original histology

	**Pathology review**						
**Original histology**	**Adeno-**	**SCC**	**UC**	**LC**	**BAC**	**Not available**	**Total**
Adeno-	54	1	0	1	0	22	78
SCC	1	8	0	1	0	1	11
BAC	1	0	0	0	0	0	1
Others	3	0	1	1	0	0	5
PD	1	1	1	4	0	0	7
NOS	3	0	0	3	0	2	8
							
Total	63	10	2	10	0	25	110

Adeno-=adenocarcinoma; SCC=squamous cell carcinoma; BAC=bronchioloalveolar carcinoma; PD=poorly differentiated carcinoma; UC=undifferentiated carcinoma; NOS=non-small-cell carcinoma, not otherwise specified.

Not available: slides not available for review.

**Table 3:** Response rates by smoking status and gender

**Status**	**Partial**	**Stable**	**Progressive**	**ORR (%)**
All patients (*n*=110)	35	15	60	32
Nonsmoker (*n*=66)	31	10	25	47
Smoker (*n*=44)	4	5	35	9
Female nonsmoker (*n*=48)	25	8	15	52
Male nonsmoker (*n*=18)	6	2	10	33
Female smoker (*n*=10)	0	2	8	0
Male smoker (*n*=34)	4	3	27	12
Chinese (102)	34	13	55	33
Malay (5)	1	2	2	25
Indian	0	0	3	0

ORR=overall response rate.

**Table 4:** Logistic regression analysis of response to Iressa

	**Coefficient (s.e.)**	**Odds ratio**	**95% confidence interval**	***P*-value**
*Gender*				0.42
Female (*n*=58)	0.42 (0.52)	1.53	0.55, 4.24	
Male (*n*=52)	—	1	—	
				
*Smoker*				0.005
No (*n*=66)	1.80 (0.64)	6.08	1.72, 21.43	
Yes (*n*=44)	—	1	—	
				
*Age in years*				0.25
<60 (*n*=65)	−0.57 (0.49)	0.57	0.22, 1.49	
⩾60 (*n*=45)	—	1	—	
				
*No. of lines of chemotherapy*				0.62
0–1 (*n*=32)	−0.26 (0.52)	0.77	0.28, 2.15	
⩾2 (*n*=78)	—	1	—	
				
*ECOG status*				0.53
0–1 (*n*=79)	0.34 (0.54)	1.40	0.48, 4.07	
2–4 (*n*=31)	—	1	—	
				
*Squamous cell histology*				0.99
Yes (*n*=11)	−19.55 (11737.8)	<0.001	0, ∞	
No (*n*=99)	—	1	—	
				
Constant	−2.01 (0.75)	0.13	—	0.007

s.e.=standard error of regression coefficient.

**Table 5:** Cox regression analysis of potential factors predictive of overall survival (defined as period from date Iressa started till last follow-up date or death)

	**Coefficient (s.e.)**	**Hazards ratio**	**95% confidence interval**	***P*-value**
*Gender*				
Female (*n*=58)	−0.04 (0.29)	0.96	0.54, 1.70	0.88
Male (*n*=52)	—	1	—	
				
*Smoker*				
No (*n*=66)	−0.12 (0.31)	0.88	0.48, 1.63	0.69
Yes (*n*=44)	—	1	—	
				
*Age in years*				
<60 (*n*=65)	0.14 (0.30)	1.15	0.64, 2.08	0.63
⩾60 (*n*=45)	—	1	—	
				
*No. of lines of chemotherapy*				
0–1 (*n*=32)	−0.29 (0.31)	0.75	0.41, 1.38	0.36
⩾2 (*n*=78)	—	1	—	
				
*ECOG status*				
0–1 (*n*=79)	−0.86 (0.27)	0.42	0.25, 0.72	0.002
2–4 (*n*=31)	—	1	—	
				
*Squamous cell histology*				
No (*n*=99)	−0.27 (0.45)	0.77	0.32, 1.84	0.55
Yes (*n*=11)	—	1	—	
				
*Response*				
SD/PD (*n*=75)	1.09 (0.34)	2.98	1.54, 5.77	0.001
CR/PR (*n*=35)	—	1	—	

**Table 6:** Cox regression analysis of potential factors predictive of overall survival (defined as period from date of diagnosis till last follow-up date or death)

	**Coefficient (s.e.)**	**Hazards ratio**	**95% confidence interval**	***P*-value**
*Gender*				
Female (*n*=58)	−0.04 (0.29)	0.96	0.54, 1.70	0.88
Male (*n*=52)	—	1	—	
				
*Smoker*				
No (*n*=66)	−0.12 (0.31)	0.88	0.48, 1.63	0.69
Yes (*n*=44)	—	1	—	
				
*Age in years*				
<60 (*n*=65)	0.14 (0.30)	1.15	0.64, 2.08	0.63
⩾60 (*n*=45)	—	1	—	
				
*No. of lines of chemotherapy*				
0–1 (*n*=32)	−0.29 (0.31)	0.75	0.41, 1.38	0.36
⩾2 (*n*=78)	—	1	—	
				
*ECOG status*				
0–1 (*n*=79)	−0.86 (0.27)	0.42	0.25, 0.72	0.002
2–4 (*n*=31)	—	1	—	
				
*Squamous cell histology*				
No (*n*=99)	−0.27 (0.45)	0.77	0.32, 1.84	0.55
Yes (*n*=11)	—	1	—	
				
*Response*				
SD/PD (*n*=75)	1.09 (0.34)	2.98	1.54, 5.77	0.001
CR/PR (*n*=35)	—	1	—	

**Table 7:** Common toxicities

**Toxicity profile**	**Grade 1 (%)**	**Grade 2 (%)**	**Grade 3 (%)**	**Grade 4 (%)**
Rash	29 (26)	5 (5)	3 (3)	1 (1)
Eyes	8 (7)	0	0	0
Diarrhoea	10 (9)	0	0	0
Asthenia	1 (1)	2 (2)	0	1 (1)
Leucopenia	1 (1)	1 (1)	0	0
Thrombocytopenia	0	0	1 (1)	0
Anaemia	17 (16)	5 (5)	1 (1)	0
Bilirubinemia	4 (4)	1 (1)	0	0
Transaminitis	13 (12)	3 (3)	1 (1)	5 (5)
